# Cryogenic electron tomography reveals helical organization of lipoprotein lipase in storage vesicles

**DOI:** 10.1126/sciadv.adx8711

**Published:** 2025-08-06

**Authors:** Kathryn H. Gunn, Anna Wheless, Thomas Calcraft, Mark Kreutzberger, Kareem El-Houshy, Edward H. Egelman, Peter B. Rosenthal, Saskia B. Neher

**Affiliations:** ^1^ Department of Biochemistry and Cell Biology, Stony Brook University, Stony Brook, NY 11790, USA.; ^2^ Department of Biochemistry and Biophysics, University of North Carolina at Chapel Hill, Chapel Hill, NC 27599, USA.; ^3^ Structural Biology of Cells and Viruses Laboratory, The Francis Crick Institute, 1 Midland Road, London NW1 1AT, UK.; ^4^ Department of Biochemistry and Molecular Genetics, University of Virginia, Charlottesville, VA 22908, USA.

## Abstract

Lipoprotein lipase (LPL) is a triglyceride lipase that is contained in intracellular vesicles in an inactive storage form before secretion, but the precise structural details have not yet been resolved. Using cryo–electron tomography (cryo-ET), we observe that LPL exists inside of storage vesicles as a filament with an 11-nanometer diameter and is packed in these vesicles in two distinct patterns. Next, we solved a 4.2-Å resolution cryo–electron microscopy (cryo-EM) structure of this 11-nanometer LPL filament using purified protein. The filament is made of repeating pairs of LPL molecules with occluded active sites, rendering the LPL inactive. The comparison of the in situ subtomogram average and the in vitro cryo-EM structure indicates that the previously uncharacterized physiological storage form of LPL is an inactive filament.

## Teaser

Cryo-ET and cryo-EM show that lipoprotein lipase forms an inactive, filamentous storage structure in vesicles.

## INTRODUCTION

Many metabolic enzymes can self-assemble into large, filamentous polymers; this phenomenon has been observed both in vitro and in cells. Filamentation can either up-regulate or down-regulate enzyme activity, and this type of regulation has a unique biological role (*1*). Assembly, and disassembly, of enzyme polymers induce changes in enzyme activity in response to environmental cues far more quickly than transcriptional or translational changes could permit. Roughly 20 metabolic enzymes are known to reversibly assemble into filaments (*2*). Notable examples include yeast glucokinase, which is inhibited by polymerization, and inosine monophosphate dehydrogenase, which is activated by polymerization (*3*, *4*).

Using cryo–electron microscopy (cryo-EM), we previously solved a structure of the enzyme lipoprotein lipase (LPL) assembled into an inactive helical filament with a 25-nm outer diameter (*5*). LPL is a secreted lipase that is active in the blood, where it is the primary enzyme that hydrolyzes triglycerides from circulating lipoproteins such as chylomicrons. Before secretion, LPL is stored in sphingomyelin-rich vesicles, and insulin signals the trafficking of these secretory vesicles out of cells and into circulation (*6*, *7*). Earlier studies reported that at high concentrations, LPL was present in a “cryptic” or inactive storage form in secretory vesicles (*8*, *9*). We used super resolution microscopy to show that endogenous LPL forms filamentous structures inside vesicles. This microscopy showed LPL filaments concentrated near the vesicle membranes, likely due to association with the transmembrane heparan sulfate proteoglycan (HSPG), syndecan-1 (SDC1). When we made mutations to abolish a key interface in the LPL filament, we no longer observed the ring-like LPL distribution in vesicles (*5*). The storage of high concentrations of LPL in an ordered but inactive form presents many potential biological advantages, including preventing lipase activity before secretion, avoiding the aggregation of concentrated LPL, and quick postprandial delivery of lipase to the bloodstream. However, the precise structure of the LPL condensate in cells remains mysterious due the scale differences between cryo-EM and super resolution microscopy.

In this study, we address the structure of LPL inside of vesicles using a combination of cryo–electron tomography (cryo-ET) and cryo-EM to show both in situ and in vitro structures of helical assemblies of LPL. A defining challenge of cryo-ET is to identify the macromolecule of interest within a crowded physiological environment. Some strategies currently used to identify proteins of interest include applying correlative light electron microscopy (CLEM) in cells or directly identifying proteins by attaching a high contrast tag for detection in tomograms (*10*). Here, we took a unique approach to identify LPL in its physiological environment by purifying LPL-containing vesicles from cells and marking these vesicles using SDC1, the HSPG that recruits LPL for packaging in the trans-Golgi (*11*).

Subtomographic averaging of filaments within LPL-containing vesicles revealed an 11-nm-diameter hollow filament. Previously, we had observed two diameters of LPL filament (25 and 11 nm) in vitro. In our previous cryo-EM work, we found that the 25-nm LPL filament was enriched on the grids, but we observed a small number of smaller 11-nm-diameter filaments. Building off these in situ observations, we returned to cryo-EM and found conditions to enrich the 11-nm LPL filament on grids and solve a 4.2-Å resolution structure. The higher resolution cryo-EM structure reveals that the 11-nm-diameter LPL helix is assembled from inactive dimers of LPL that are very similar to those that are the basis for the 25-nm helix. We then were able to compare the cryo-EM structure to the subtomogram average from the cryo-ET data to show that there is agreement between the two volumes. Thus, LPL is stored within vesicles as filaments, which are 11 nm in diameter, and composed of inactive LPL dimers.

## RESULTS

### Localization of LPL within secretory vesicles

**Fig. 1.**
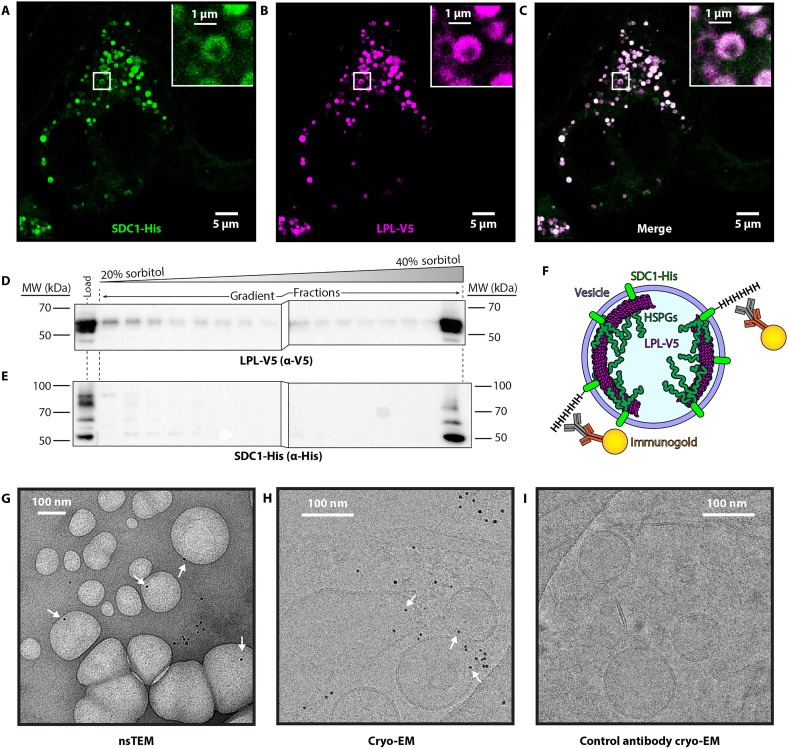
Expression, isolation, and labeling of LSVs for cryo-ET. (**A**) HEK293 cells transiently transfected with both SDC1 and LPL were imaged using STED super resolution microscopy. Immunofluorescence staining for the hexahistidine (His) tag on SDC1 (green) shows localization of the SDC1 to vesicle membranes. (**B**) Simultaneously, LPL tagged with V5 was probed with anti-V5 (purple), and LPL also displayed localization to vesicles. (**C**) The merged image of (A) and (B) shows overlap between SDC1 and LPL in white. Cells transfected as the ones imaged in (A) to (C) were gently lysed to allow isolation of intact vesicles from the cells. (**D **and **E**) Western blot analysis of sorbitol gradients of the isolated vesicles shows that the majority of the LPL/SDC1-containing vesicles migrate to the bottom of the gradient and are enriched in the last fraction. Following enrichment of LPL-containing vesicles using the gradient, we leveraged the external facing SDC1 His-tag to attach gold immunolabels to the vesicles. (**F**) A diagram illustrating our labeling strategy to identify LPL-containing vesicles. SDC1 HSPGs target LPL into specific secretory vesicles, but by using the cytoplasmic side of SDC1 as a target, we can label it without disrupting the membrane or affecting the internal LPL structure. (**G**) Negative stain TEM image assessing the presence of immunogold labels on anti-His–labeled vesicles. White arrows indicate label locations on vesicles. (**H**) Cryo-EM micrograph of anti-His– and immunogold-labeled vesicles, white arrows indicate overlap of immunogold and vesicles. (**I**) Vesicles treated with control antibody and immunogold do not show overlap of immunogold and vesicles. Scale bars, 5 μm [(A) to (C)] and 100 nm [(G) to (I)]. MW, molecular weight.

We previously found that LPL inside of secretory vesicles was atypically distributed. Instead of being distributed evenly throughout intracellular vesicles, LPL was concentrated on the inner walls of LPL storage vesicles (LSVs), appearing in a ring-like shape that indicates nonrandom localization (*5*). These vesicles were also marked with SDC1, which recruits LPL into vesicles in the trans-Golgi with its heparan sulfate chains (*11*). We found that the ordered, ring-like distribution of LPL could be recapitulated in human embryonic kidney (HEK) 293 cells by transiently expressing both LPL and SDC1 (Fig. 1, A to C). However, super-resolution microscopy could only reveal LPL’s location, not its molecular form. We therefore turned to cryo-ET to determine the structural form of LPL present within LSVs. We isolated large quantities of LSVs from HEK293 cells expressing epitope-tagged LPL and SDC1. The epitope tags did not affect the filament distribution of LPL in the vesicles compared to our previous experiments (*5*). We then isolated a general population of vesicles from cell lysate using centrifugation and identified fractions containing LPL by Western blotting to obtain samples enriched in LSVs for imaging (Fig. 1, D and E) (*7*). Full gels for the gradient fractionation are available as fig. S1.

To identify LPL-containing vesicles by cryo-ET, we took advantage of the cytoplasmic-facing C-terminal domain of SDC1 to tag the vesicles with a hexahistidine epitope tag. We then labeled the SDC1 using immunogold to enable identification of LPL-containing vesicles without disrupting the inner contents of the vesicles, as shown in Fig. 1F. SDC1’s N-terminal domain faces the inside of the vesicle and contains the heparan sulfate moieties that recruit LPL into vesicles (*11*). We used negative stain transmission electron microscopy (nsTEM) to validate the successful isolation and labeling of LSVs (Fig. 1G). We next plunge froze the vesicles onto cryo-grids for screening. This revealed that the heavy gold labels were present in the sample and identifiable at lower magnifications for target detection (Fig. 1H). To overcome sparse labeling, we used high ratios of the immunogold labels, which led to the presence of some free gold labels. To confirm that the gold labels on the vesicles were not free labels that coincidentally localized near vesicle membranes, we performed a control using mouse immunoglobulin G (IgG) antibody instead of the anti-histidine tag antibody recognized by the immunogold antibodies. In these control images, while we did see free immunogold, we did not observe any immunogold associated with the vesicles (Fig. 1I).

### Imaging LPL vesicles using cryo-ET

To further characterize the LSVs, we performed cryo-ET. We collected preliminary tomograms and screened the grids for intact vitreous ice using a Talos Arctica equipped with a Gatan K3 camera. Primary data collection was performed with a Titan Krios equipped with a Falcon4i camera. At medium magnification, ~75 locations with vesicles that appeared immunogold-labeled were selected for tomography. Tomograms were collected using SerialEM from −60 to +60 tilt, in steps of 3°, using a dose symmetric pattern. The tomograms were preliminarily reconstructed using Warp (*12*) to check the proximity of the immunogold labels to the vesicle membrane before moving on to further reconstruction. In total, 77 tomograms were collected, and of these, 19 tomograms contained immunogold-labeled vesicles. These 19 tomograms were reconstructed with IMOD (*13*). To facilitate visualization and particle identification, the tomograms were then denoised with Topaz (*14*) and corrected using IsoNet (*15*). In these final reconstructions, we selected those vesicles where the immunogold labels were within 20 Å of the vesicle membrane for our final dataset because this is approximately the maximum distance that two antibodies would place the gold away from the membrane. In total, we identified 40 vesicles with immunogold labeling and manually examined each vesicle. Putative LPL helical filaments were identified in all SDC1 gold-labeled vesicles (fig. S2). We observed that the filaments were arranged in vesicles in two different configurations, with ~50% of the vesicles falling into each category.

**Fig. 2.**
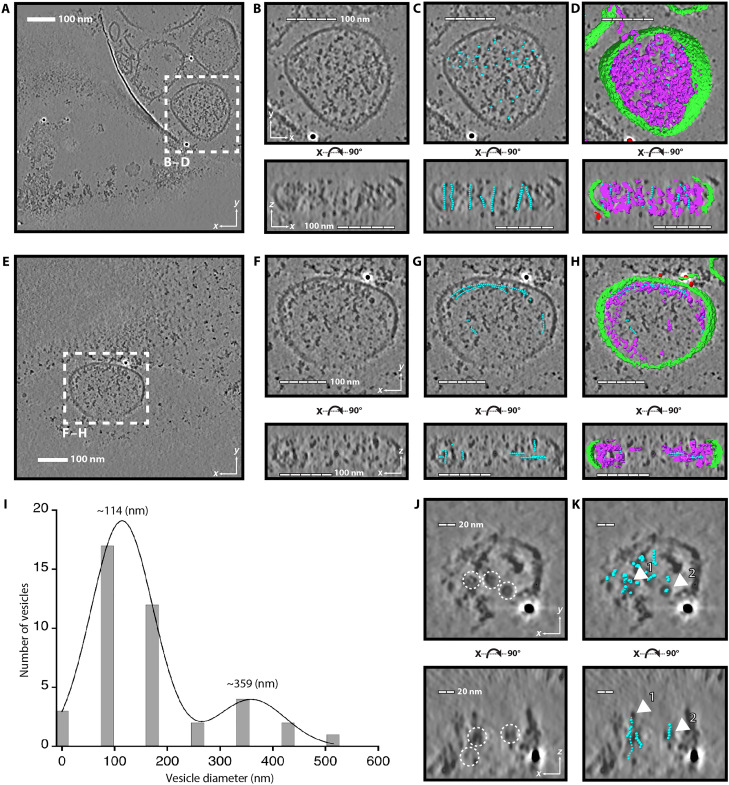
Analysis of LSVs by cryo-ET. (**A**) A 2D slice of a reconstructed, denoised, and corrected tomogram shows potential LSVs in vitreous ice. (**B**) Zoom of the vesicle from (A). The top image is a slice viewed in the *XY* plane. The lower image is a slice from the *XZ* plane. Scale bars, 100 nm [(B) to (D) and (F) and (G)] in 20-nm segments. (**C**) Locations of potential LPL filaments were manually picked. Each cyan sphere indicates the location of one particle. The filaments in this vesicle are packed in one direction, as seen in the *XZ* plane. (**D**) We trained the Dragonfly neural net to segment tomograms. Immunogold labels are red, plasma membranes are green, and LPL filaments are purple. The segmentation shows LPL filaments located throughout the vesicle, consistent with particle picking (cyan). (**E**) A 2D tomogram slice illustrating another LSV. (**F**) Zoom of the vesicle outlined in (E), viewed from the *XY* and *XZ* planes. (**G**) Locations of manually picked particles for LPL filaments (cyan). Filaments are not aligned in the same plane. (**H**) Vesical segmentation shows that LPL filaments (purple) are clustered near the plasma membrane (green), agreeing with manual picking (cyan). (**I**) Approximate LSV diameter binned and fit by a double Gaussian. (**J**) Zoomed in LSV illustrates manual particle picking. Potential filaments were identified because they appeared as a circle [top image (*XY*), dashed circles] or parallel lines [lower image (*XZ*), dashed circles]. Filaments were aligned in a single direction, so circles appear in *XY* and parallel lines in *XZ*. Scale bars, 20 nm [(J) and (K)] in 10-nm segments. (**K**) Overlay of particle picks (cyan) fits data from 2D slices. Filaments labeled 1 and 2 in the top image (*XY*) are also labeled in the lower image (*XZ*).

Approximately half of the LSVs were packed with filaments throughout the vesicle. In some cases, this packing created filaments that were all aligned in the same direction (Fig. 2, A and B). We will refer to this arrangement as “packed” vesicles. Manually picked filament locations (cyan) are distributed throughout the packed vesicle (Fig. 2C). To generate complete three-dimensional (3D) renderings of the packed vesicle and its contents, we used Dragonfly (*16*) to train and implement a neural net segmentation that could identify putative LPL filaments, plasma membrane, and fiducial labels. The 3D segmentation (Fig. 2D) highlights the distribution of putative LPL filaments (purple) throughout the vesicle and was used to substantiate the manually picked locations.

In the second configuration of helices inside the LSVs, filaments segregated to areas near the plasma membrane, leaving the center of the vesicles empty (Fig. 2, E to H). This pattern mimics our observations of ring-like structures seen with immunofluorescence microscopy (Fig. 1A), so we will refer to these vesicles as “ring like.” The filaments in ring-like vesicles are sparser than the ones packed vesicles and do not create the semi-ordered packing pattern, appearing both parallel and perpendicular to other filaments. We also observe that ring-like filaments can be longer than those in the packed vesicles. This could be due to the orientation of the vesicles within the ice. Larger vesicles were compressed during grid freezing, distorting their spherical shape. Most packed vesicles were aligned so that the filaments traveled in the *Z* plane, which is compressed by grid preparation, and this compression could artificially shorten their length. When we analyzed the vesicle diameters by taking three diameter measurements of each vesicle in the *XY* plane and averaging them, we found vesicle size ranged from ~50 to 500 nm in diameter, with the caveat that the field of view and ice thickness placed upper limits on our samples. The distribution of vesicle diameters was best fit with a double Gaussian with peaks at ~114 and ~359 nm (Fig. 2I). Although there are variations (fig. S2), the larger diameter and, therefore, more compressed vesicles trend toward the ring-like filament distribution.

The smallest vesicle in the data has a diameter of ~50 nm, and due to the thinness of the sample, most clearly illustrates the putative LPL filaments within (Fig. 2, J and K). This vesicle falls into the packed vesicle category, with filaments distributed throughout the vesicle. In the *XY* plane, we see that the filaments visible in a 2D slice all appear as small rings (indicated by dashed circles). The filaments are oriented with their long axis traveling along the *Z* plane. When we rotate the tomogram 90° and take another 2D slice, we can see the *Z* length of the same filaments appear as two parallel lines from either side of the filament (dashed circles in Fig. 2J and additional example shown in movie S1). The analysis of the filament locations (cyan) reveals the same filaments (labeled 1 and 2, respectively, in Fig. 2K) in both the *XY* and *XZ* planes.

The examination of these tomograms revealed that the filament structures did not directly correlate to the high-resolution cryo-EM structure of the LPL filament we previously solved, which has a diameter of 25 nm (*5*). The diameter of the filaments in the tomograms is ~11 nm. Although this diameter did not match the high-resolution structure of the 25-nm helix, it did match the smaller LPL filament we observed in both cryo-EM and negative stain data. We therefore set out to perform subtomogram averaging of the LPL in vesicles and also solve the high-resolution structure of the 11-nm LPL filament observed in vitro.

### Subtomogram averaging of LSV filaments

**Fig. 3.**
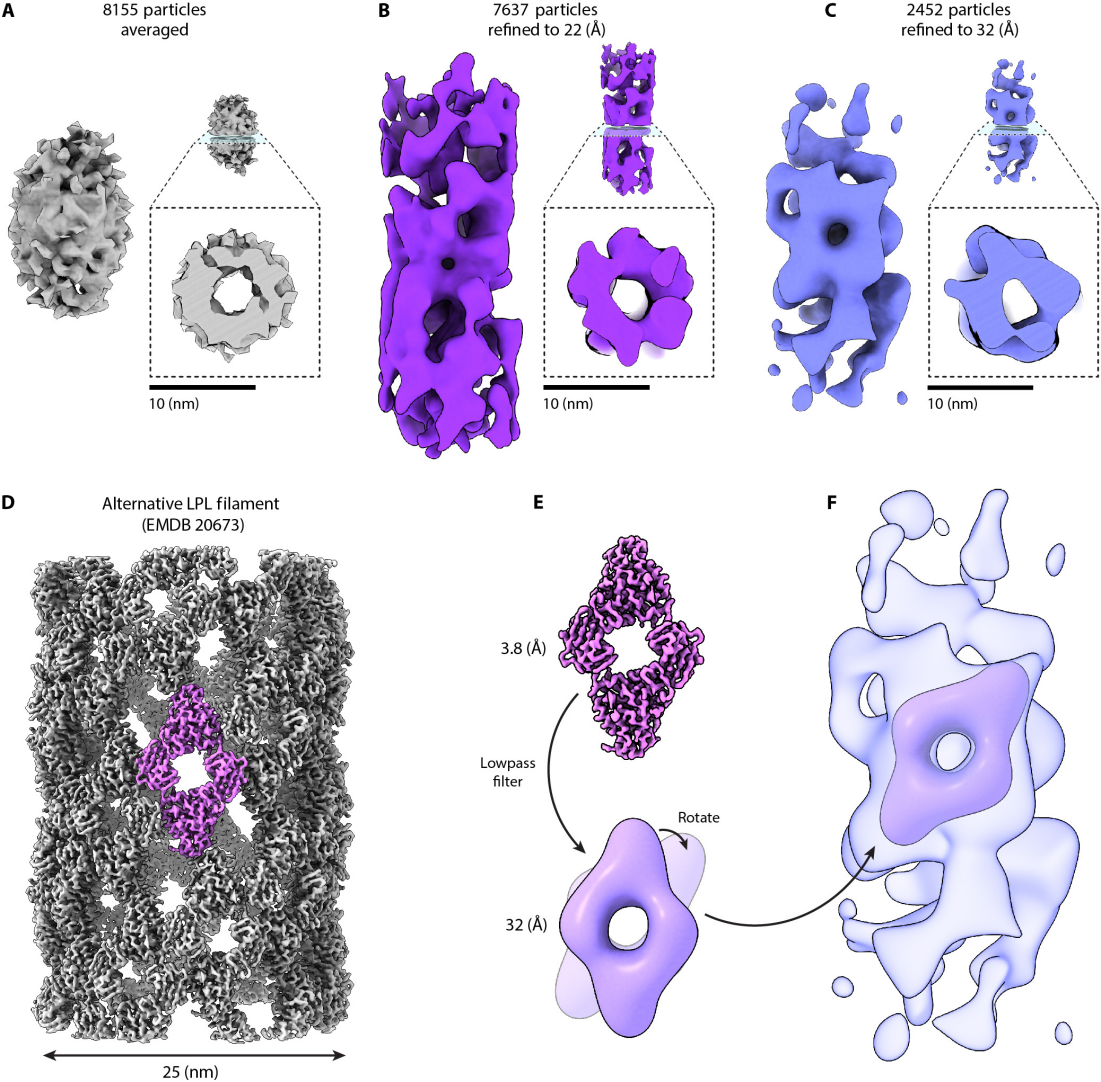
Subtomogram averaging of LSV filaments. (**A** to **C**) Left: LPL filament volumes from various stages of processing. Right: Cross section of the middle of the accompanying volume showing a hollow interior and consistent diameter of ~11 nm. (A) Average of the 2D projections of all particles picked by eye in ArtiaX (8155 particles), reconstructed in RELION without alignment or symmetry. (B) Refined average of 7637 particles aligned without symmetry after removal of lipid membrane and low-quality particles, rendered at threshold level 0.015. (C) Refined average of 2452 particles isolated from straight filaments aligned without symmetry parameters, rendered at threshold level 0.0206. (**D**) EMDB entry 20673, the 3.8-Å resolution map of the 25-nm LPL helix determined by cryo-EM (*5*), used as a reference for the current study (threshold level of 0.0137). (**E**) Top: Isolated dihedral LPL dimer volume from (D) using volume mask tool in ChimeraX. Bottom: Dimer volume subsequently lowpass filtered to 31 Å with RELION Image Handler to match the estimated resolution of the reconstruction in (C) and rotated 35° about the *z* axis. (**F**) Filtered and rotated dihedral dimer from the large LPL helix from (E) aligned to the small LPL filament reconstruction from (C).

To identify particle locations for subtomogram averaging, we manually picked filament locations in ChimeraX using the ArtiaX add on (*17*, *18*). This allowed us to delineate the filament and then use ArtiaX to split the filament into a series of particle locations 36 Å apart, with random alignment of their *X* and *Y* direction around the *Z* axis to help address the missing wedge (*15*). We loaded these 8155 particle locations into RELION 4 (*19*) along with the motion-corrected micrographs and tomogram alignment data. Initial averaging of these particles revealed a hollow cylinder of ~11-nm diameter (Fig. 3A). After excluding particles classified as lipid bilayers, we continued subtomogram alignment and refinement and produced a low-resolution map of the putative LPL filaments from 7637 particles (Fig. 3B). With this 22-Å resolution volume, we were able to visualize general characteristics of the oligomer. The outer diameter matched what we had estimated from preliminary negative stain EM, but the overall architecture did not closely resemble the net-like, repeating diamond pattern of the 25-nm LPL filament we solved previously (*5*). Many of the filaments near the membrane are bent in the vesicles. Even slight bending severely disrupts the filament’s helical symmetry, so we isolated a particle subset from straight filaments for additional processing (2452/7637 or ~1/3 of the particles, workflow shown in fig. S3). This resulted in a cryo-ET map with an overall resolution of 32 Å (Table 1). The resulting volume (Fig. 3C) retains its characteristic inner and outer diameters and also contains a region that closely matches the shape and size of the dihedral LPL dimer from the 25-nm LPL helix described previously (Fig. 3D), albeit in a slightly different orientation (Fig. 3, E and F). To better understand these differences, we next set out to determine a high-resolution structure of the 11-nm LPL filament.

**Table 1.** Refinement statistics for filament map and model resolved by cryo-EM helical reconstruction (HR) and filament map resolved by cryo-ET subtomogram averaging (STA). Statistics for the cryo-EM map (left column) were obtained from cryoSPARC, and model statistics were obtained from PHENIX Real-Space Refinement. Statistics for the subtomogram average map were obtained from the RELION tomography package. Resolution values are given in Ångstroms (Å). PDB, Protein Data Bank; EMDB, Electron Microscopy Data Bank; EMPIAR, Electron Microscopy Public Image Archive; NA, not applicable.

	Cryo-EM HR	Cryo-ET STA
**Data collection and processing**		
Magnification	45,000	64,000
Voltage (kV)	200	300
Pixel size (Å)	0.876 Å	1.965 Å
Total dose (e^−^/Å^2^)	58	101
Number of particles	45,000	2,452
*B* factor	−220	1,000
**Map resolution (Å)**		
Model:Map FSC (0.5)	4.6	NA
Map:Map FSC (0.143)	4.2	20
Map:Map FSC (0.5)	4.7	32
*d*_99_	4.5	NA
Symmetry	D1	C1
**Model refinement**		
Model to map CC	0.77	NA
Clash score, all atoms	8.88	NA
Ramachandran favored (%)	88.16	NA
Ramachandran outliers (%)	0	NA
Rotamer outliers (%)	0	NA
Cß deviations >0.25 Å	0	NA
**RMS deviations**		
Bond (Å) (# > 4σ)	0.004 (0)	NA
Angles (°) (# > 4σ)	0.596 (0)	NA
**Deposition IDs**		
PDB ID	9NRN	NA
EMDB ID	EMD-49738	EMD-49737
EMPIAR ID	NA	EMPIAR-12625

### High-resolution cryo-EM structure of 11-nm-diameter LPL filament

**Fig. 4.**
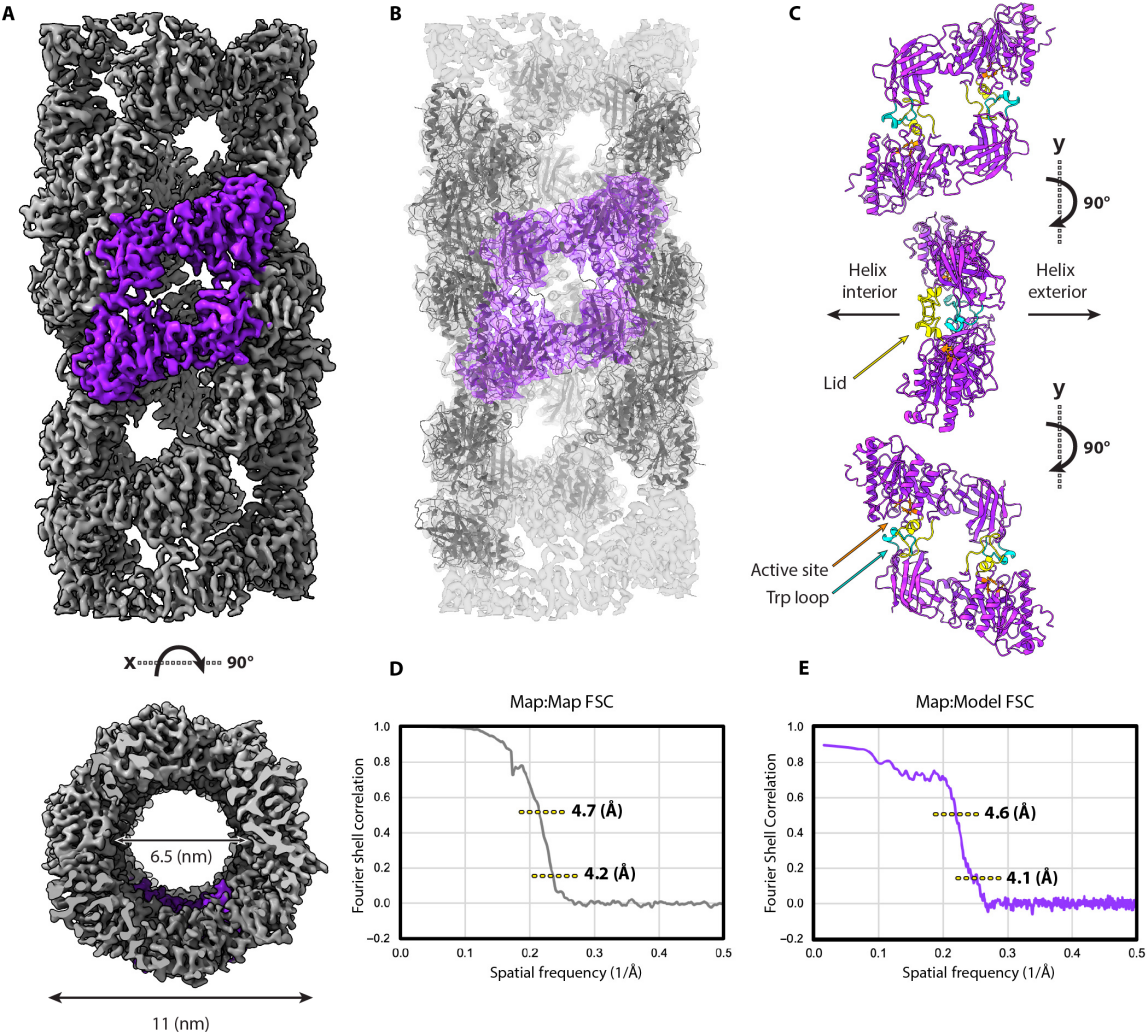
Map and model of the 11-nm LPL filament resolved by cryo-EM helical reconstruction. (**A**) Map of the 11-nm LPL filament resolved by cryo-EM. The region highlighted in purple is the dihedral LPL dimer in a head-to-tail arrangement that comprises the asymmetric subunit of the helical filament. (**B**) Model (space-filling view) of the 11-nm LPL filament showing the dihedral LPL dimer in purple, as in (A). (**C**) Model (ribbon view) of the dihedral LPL dimer that is the base repeating unit of the filament, rotated about the vertical axis. Residues in orange are part of the tryptophan loop that is responsible for substrate binding by LPL. The LPL lid domain is shown in yellow, and the active site catalytic triad is shown in blue. (**D**) Map:Map FSC curve for the 11-nm filament solved by cryo-EM helical reconstruction. The FSC(0.143) resolution of the masked volume is 4.2 Å and the FSC(0.5) resolution is 4.7 Å. (**E**) Map:Model FSC curve for the masked 11-nm filament solved by cryo-EM. The FSC(0.143) value is 4.1 Å, and the FSC(0.5) value is 4.6 Å. FSC, fourier shell correlation.

We tested a variety of buffer conditions using nsTEM to increase the proportion of 11-nm-diameter LPL filaments relative to 25-nm filaments. We found that using 10 mM Hepes (pH 7.2) enriched the 11-nm filaments on grids for nsTEM (fig. S4, A and B). However, when applied to grids for cryo-EM, the same buffer only contained 25-nm LPL filaments. We hypothesized that the continuous support on the negative stain grid versus the cryo-EM grid could favor the 11 nm form. We therefore froze cryo-EM samples using grids with a 2-nm continuous carbon film to better mimic the negative stain grid environment and successfully enriched the 11-nm LPL filament (fig. S4C). Using these conditions, we collected data using a Talos Arctica equipped with a K3 direct detector and analyzed the data in cryoSPARC (fig. S5) (*20*). We were able to determine a 4.2-Å structure of the 11-nm LPL filament and build a molecular model into the cryo-EM density using a monomer from the 25-nm filament as a starting model (Fig. 4, A to D; Table 1; and fig. S6). As was seen in the 25-nm LPL filament, the base subunit of the 11-nm LPL helix is a dihedral dimer of LPL. However, the helical symmetry differs between the thinner and wider filaments, leading to differences in the helical interaction interfaces (figs. S5 and S7). As we previously observed, the dihedral dimer is arranged head-to-tail, such that the substrate recognition domain of one LPL occludes the active site of the other LPL (Fig. 4C and fig. S7). This arrangement buries hydrophobic residues and creates a highly favorable interface (table S1 and fig. S8). We have previously shown that LPL in this conformation is inactive (*5*). Another difference between the structures is the resolution of the flexible lid of LPL, which was poorly resolved in the 25-nm-diameter structure. Although the resolution of the lid was lower than the rest of the map in the 11-nm-diameter structure, it was high enough to allow it to be modeled in this dataset (Fig. 4C and fig. S6 as well as local resolution map in fig. S9). Thus, the LPL lid domain may be less mobile in the narrower interior of the thinner filament.

### Comparison of 11-nm LPL filament helical reconstructions

**Fig. 5.**
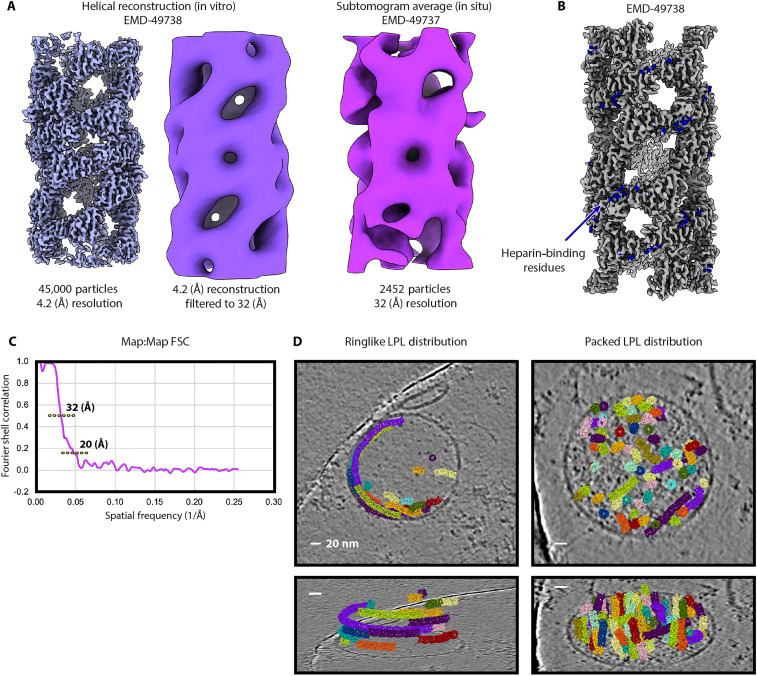
Features of 11-nm LPL filament in vitro and in situ. (**A**) Left: Unfiltered, 4.2-Å resolution helical reconstruction of LPL filament refined from 45,000 particles, contour level of 0.163. Center: The same map as on the left lowpass filtered to 32-Å resolution for comparison to the STA volume on the right, viewing threshold 0.0382. Right: Subtomogram average reconstructed without symmetry from 2452 particles picked from straight filaments, contour level of 0.110. Resolution reported by RELION 3DRefine was 31 Å, and the final map has a FSC(0.5) resolution of 32 Å. (**B**) Cryo-EM volume showing known LPL heparan-binding residues in dark blue (residues 309, 310, 312, 326, 327, 433, 435, 437, 443, and 444 of bovine LPL). (**C**) FSC between the two randomized halfmaps (map:map FSC). FSC(0.143) resolution is 20 Å, and the FSC(0.5) resolution is 32 Å. (**D**) Cryo-EM helical map used as a surface model for the filaments selected from representative tilt series for ring-like (left, tilt series 44) and packed (right, tilt series 40) filament distribution patterns [(A) and (B) rendered in ChimeraX and (D) rendered in ArtiaX add-on for ChimeraX].

We next compared the helical cryo-EM structure with the subtomogram average. The subtomogram average and high-resolution cryo-EM structures both have similar outer diameters and hollow centers (Figs. 3C and 4A). The characteristic spaces in the helical lattice seen in the 4.2-Å helical cryo-EM map partially align with the subtomogram average of particles from straight filaments (Fig. 5A). This is visible when the cryo-EM structure is filtered to the reported resolution of the subtomogram average. Although this cryo-ET map was produced from about 5% of the chosen particles for the cryo-EM structure and was processed without any application of symmetry, the diamond-shaped volume of a dihedral LPL dimer—a 100-kDa object—is visible in the center (Figs. 3E and 5A). The subtomogram average lacks the clear global symmetries that were identified in helical reconstruction. Without the resolution required to determine helical and point group symmetries, those parameters could not be applied in processing to aid reconstruction. The symmetry parameters from the helical structure did not improve the subtomogram average. The tomography was performed on human cell vesicles expressing human LPL, while the cryo-EM images are of bovine LPL, a 94% identical ortholog that is more readily purified. A high-resolution structure of human LPL filaments may have revealed different helical symmetry. One contributing factor to the limited resolution in the subtomogram average is the relatively small number of particles, which had to be manually selected from low-contrast images. Another factor is heterogeneity within that small dataset from both the slight curvatures of the filaments and the likely presence of other molecules, specifically the heparan chains on SDC1, which are highly heterogeneous. Heparan chains have an average molecular weight of ~30 kDa and can stretch up to 150 nm in length (*21*), and each SDC1 has three sites for heparan attachment (*11*). We have previously shown that heparan provides stability against dissolution of the LPL filament (*5*). The known heparan-binding residues of LPL are located on outside of the filament (Fig. 5B). Notably, the 2452 particles that produced the clearest subtomogram average were from straighter filaments, which tend to be in the vesicle centers and not near the membranes where HSPGs are anchored. It is likely that HSPGs are binding to the small LPL filaments inside vesicles, especially near the outer edges, and may be stabilizing them.

The differences between these two packing patterns can be most easily seen when viewed in the context of the vesicle. We can see trends in localization and density that comprise both the packed arrangement and the ring-like arrangement of filaments (Fig. 5D). LPL filaments in packed vesicles are further from the membrane and more ordered, likely having less HSPG binding than filaments that are membrane adjacent. This arrangement more efficiently packs LPL into the space of the vesicle. The ring-like filament arrangement keeps filaments close to the membrane, where they can be found in groups or as solitary filaments. The LPL concentration in the ring-like pattern appears lower than in the packed pattern. This may mean that maintaining filamentous LPL structure in a ring-like arrangement relies more heavily on HSPGs, whereas the packed pattern can use the higher relative concentration of LPL to maintain filament formation.

## DISCUSSION

Here, we report cryo-ET studies of LPL from vesicles purified from their native cytoplasmic environment. Most of the cellular LPL visualized via immunofluorescence has a notable ring-like arrangement in vesicles, but some of the LPL is distributed evenly in other vesicles (Fig. 1A). These different arrangements could represent distinct pools of LPL that are held in the cell. For example, the packed vesicles could be secreted in response to insulin to quickly increase the amount of LPL in the capillaries adjacent to the adipocytes (*7*), while ring-like vesicles could represent the continuous flow of LPL for secretion. Future work looking at natively expressed LPL could reveal information about the endogenous prevalence of these two packing types.

This study demonstrates the utility of cryo-ET in bridging the space between super resolution fluorescence microscopy and cryo-EM. The in situ reconstruction of the LPL filament validates the in vitro structure’s physiological relevance and supports our explanation of the atypical distribution of LPL inside of secretory vesicles seen by fluorescence microscopy. To facilitate our cryo-ET data collection and identify LPL in vesicles, we removed the vesicles from the cellular environment, which is a limitation of our study. Future studies could use CLEM to target LSVs inside cells and image the LPL structure and its vesicular distribution within the cell, providing more context for the packing modes observed in our study. Study of cells that endogenously express LPL would also be beneficial to learn more about vesicle packing in a native environment.

Together with previous research, our findings suggest that formation of an inactive quaternary structure prevents off-target LPL activity while surrounded by cellular lipids during vesicular storage and trafficking. The regulation of enzymatic activity by filamentation is a mechanism used by other enzymes with roles in metabolism that allows enzyme activity to be tuned in response to environmental cues (*2*). The concentration dependence of the LPL helical filament we observe in vitro, suggests that upon secretion to the interstital space, LPL would experience rapid dilution, leading to dissolution of the inactive filament. This would then allow LPL to regain its capacity to bind substrate and other regulatory elements needed to facilitate its transition into the capillaries.

## MATERIALS AND METHODS

### Cell culture and microscopy

A T-Rex HEK293 stable cell line with inducible Lmf1 expression was used as previously described (*22*). Cells were cultured in Dulbecco’s modified Eagle’s medium supplemented with 10% fetal bovine serum, penicillin/streptomycin, and l-glutamine. Cells were plated in six-well plates containing 18-mm round #1.5 high accuracy coverslips. Coverslips were prepared by incubation for 5 min in 6 N HCl, rinsed in water, washed with 70% ethanol, air-dried, sterilized under ultraviolet light, and then functionalized with poly-d-lysine and fibronectin. Plasmids containing LPL-V5 and SDC1-HA-His were transfected into ~75% confluent HEK293-Lmf1 cells using calcium phosphate transfection with a plasmid ratio of 2:1 LPL:SDC1. Thirty min before transfection, tetracycline was added to the culture media to induce production of Lmf1 to aid LPL folding. Transfections were incubated overnight at 37°C. The cells were fixed using 0.05% glutaraldehyde and 2% paraformaldehyde. Cells were permeabilized with 0.1% Triton X-100 and blocked using 5% donkey serum. Fixed cells were first labeled with a goat anti-LPL antibody (R&D Systems, AF7197) at a 1:40 ratio in a humidified chamber overnight at 4°C. Cells were next incubated with chicken anti-goat labeled with Alexa 594 for 1 hour at 22°C. The cells were then labeled with a rabbit anti-HA (Cell Signaling Technology, 3724) at a 1:500 ratio in a humidified chamber overnight at 4°C. The next day the cells were incubated with goat anti-rabbit secondary labeled with ATTO 647 N (Sigma-Aldrich, 40839) at 1:100 for 1 hour at 22°C. Coverslips were mounted using antifade diamond (Invitrogen) and dried overnight at 4°C. Slides were imaged with a Leica Stellaris 8 Falcon stimulated emission depletion (STED) outfitted with a 775-nm STED laser. Images were collected with LasX software using a 100× objective. Time-gated Tau-STED was used.

### Vesicle purification

For vesicle purification, cells were cultured and transfected as described above in T75 flasks. Transfections were incubated for 36 hours at 37°C. Cells were scraped from the flasks and resuspended in ice-cold phosphate-buffered saline (PBS) with 1 mM phenylmethylsulfonyl fluoride (PMSF) and 4 mM EDTA. Cells were pelleted at 500*g* for 10 min at 4°C and then resuspended in lysis buffer [20 mM Hepes (pH 7.2), 2.5 mM EDTA, 1 mM PMSF, and 14% sorbitol). Cells were lysed by passing sequentially through a 25- and 26-gauge needle. The sample was spun at 500*g* for 10 min at 4°C to pellet the nuclear fraction and cell debris. The supernatant was transferred on top of a 20% sorbitol cushion [20% sorbitol, 20 mM Hepes (pH 7.2), and 2.5-mm EDTA] and spun at 100,000*g* for 1.5 hours at 4°C. The resulting pellet was resuspended in 150 μl of lysis buffer and incubated for 1.5 hours while rotating at 4°C with 5% goat serum for blocking. The blocked sample was layered on top of a continuous 20 to 40% sorbitol gradient with 20 mM Hepes (pH 7.2). The gradients were spun at 24,000 rpm (77,400*g*) in a Beckman SW60Ti rotor for 60 min at 4°C and then fractionated by hand. The pellet at the bottom of the gradient was resuspended in 100 μl of 5% sorbitol with 20 mM Hepes (pH 7.2). The pellet fraction was rotated at 4°C overnight with a 1:20 dilution of mouse anti-His antibody or mouse IgG control antibody. The next morning, the vesicles were layered on top of a 20% sorbitol cushion, and tubes were filled with PBS. The samples were spun at 100,000*g* for 1.5 hours at 4°C. The pellet was resuspended in 5% sorbitol with 20 mM Hepes (pH 7.2) and incubated at a 1:2 ratio with 6-nm gold-labeled goat anti-mouse antibody overnight with rotation at 4°C. The next morning, the vesicles were layered on top of a 20% sorbitol cushion, and tubes were filled with PBS. The samples were spun at 100,000*g* for 1.5 hours at 4°C. The resulting pellet of labeled vesicles was resuspended in 100 μl of 5% sorbitol with 20 mM Hepes (pH 7.2) and either immediately taken for grid preparation or flash-frozen and stored at −80°C. Vesicle purification was monitored by SDS–polyacrylamide gel electrophoresis (SDS-PAGE). Fractions were separated by SDS-PAGE and then transferred to PVDF membranes. LPL was detected with 1:1000 with mouse anti-V5 (Bio-Rad, MCA1360) overnight at 4°C. SDC1 was detected with 1:5000 with mouse anti-His (Bio-Rad, MCA1396) overnight at 4°C. The next day, the blots were incubated with secondary goat anti-mouse horseradish peroxidase (Southern Biosciences, OB1030-05) at 1:1000 for 1 hour at 22°C. Blots were developed with Advansta ECL substrate and imaged with a Bio-Rad Chemidoc. Vesicle labeling was checked using nsTEM. Vesicle samples in 5% sorbitol with 20 mM Hepes (pH 7.2) were applied to 300-mesh copper grids with standard thickness carbon (EMS, CF300-Cu-50). The grid was washed with 5% sorbitol with 20 mM Hepes (pH 7.2) and stained with 2% uranyl acetate supplemented with 5% sorbitol. Grids were imaged as described below.

### Cryo-ET grid freezing

Using a Vitrobot Mark IV, samples were frozen on R1.2/1.3 200-mesh Quantifoil grids—with copper support, at 4°C, 100% humidity, −10 blot force, with no wait time—and blotted for 4 to 5 s. A volume of 3 μl of vesicle sample was applied to the grid in 5% sorbitol with 20 mM Hepes (pH 7.2).

### Cryo-ET data collection

Cryo-ET data were collected through the National CryoET network with the Midwest Center for cryo-ET. Grids were imaged with a 300-kV Titan Krios equipped with a Falcon 4i camera. Tomograms were collected using serialEM (*23*). Locations for data collection were picked manually, trying to select vesicles near the 6-nm gold labels. In total, 77 tomograms were collected. Tomograms were collected in steps of 3° using a dose symmetric method starting at 0° and progressing to ±60°. The magnification for data collection was ×64,000, giving a pixel size of 1.965 Å. The total dose was 101 e−/Å^2^.

### Tomogram reconstruction

Tomograms were reconstructed from the collected tilts using the gold labels in the samples as fiducials with IMOD (*13*). Weighted back projection was the method for tomogram assembly. From the 77 collected tomograms, we found 19 contained labeled vesicles, which we defined as a 6-nm gold within 25 nm of the plasma membrane. A distance of 25 nm was chosen as the maximum due to the approximated size of the two antibodies (10 nm each) combined with the size of the 6-nm gold bead. To enhance contrast and make particle picking easier, the tomograms were then denoised with Topaz denoise 3D (*14*). Deep learning with isonet (*15*) was used to further increase the signal-to-noise ratio and predict the missing wedge. Tomograms were viewed in ChimeraX (*17*). CtfFind4 was used to estimate the CTF (*24*).

### Subtomogram averaging

Particle picking was performed in ChimeraX using the ArtiaX add-on (*17*, *18*). Filaments were created by laying points along each filament and creating a line interpolating between the points. The line models were then used to create particles separated by 36 Å and with ~30° twist around the *z* axis. The randomizing of the initial *x* and *y* direction assigned to the particle or twist of each particle was done to help alleviate the missing wedge artifacts during subsequent subtomogram averaging.

Tomograms (without contrast or signal enhancements) and particle coordinates from 19 tilt series were imported into RELION v4.0.1 for subtomogram averaging (*19*). Processing began with 8155 particles at a binning factor of four (7.860 Å/pixel). An initial unbiased model for refinement was made from first extracting pseudosubtomograms for all these particles and then averaging the 2D projections with the Tomo reconstruct particle job. Next, 3D classification was used to filter out indistinct particle images (358 particles) and particles that appeared to be lipid bilayer (160 particles). The remaining 7637 particles were iteratively refined in RELION with the 3D autorefine job, proceeding from a binning factor of four to two (3.930 Å/pixel) to unbinned (1.965 Å/pixel). Key parameters for refinement included helical reconstruction with a specified outer diameter, and no value specified for the inner diameter so that any hollow interiors of resulting filament maps would be reflective of the particles’ densities and not a result of masking. No helical symmetry or point group symmetry was applied during the processing history of the volumes in this report. No local searches of symmetry were performed because symmetric features were smaller than the reported resolution.

During processing, the resolution of the subtomogram average was not improving as expected, and we tested flipping the handedness of the data. We used a script that flipped both the *z* coordinate of the particle locations and the IMOD alignment data for the tilt series, resulting in reconstructed tomograms flipped on the *z* axis and particles of the opposite hand. This inversion significantly improved the appearance of the subtomogram average even without further refinement of the particles. This result was due to better CTF correction of the tomograms with the correct hand. The improvement caused by inversion confirmed our theory that the original handedness applied to the data was incorrect, and we proceeded with the flipped data for the rest of the data analysis.

In the flipped dataset of 7637 particles, without applying helical symmetry, aligning the particles yielded a 23-Å resolution structure as reported by RELION 3D Refinement. We theorized that the inherent curvature and heterogeneity of the filaments inside vesicles—particularly those that ran along the membrane—could be breaking the symmetry, resulting in a lack of defined features in the map.

To address the hypothesized structural heterogeneity, we took the refined particle locations and selected filaments that were not adjacent to the membrane and did not display curvature over the length of the filament. This resulted in a dataset of 2452 particles. Refining the subset of particles resulted in a map with lower resolution as reported by RELION 3D autorefine but improved map features that are more readily identifiable as an LPL oligomer. We later applied the helical symmetry parameters (rise = 23.7 Å and twist = −65.95°) that were derived from our helical cryo-EM structure (as described below). This did not result in an improvement in the structure’s features, suggesting that the helical symmetry may be different from the helical cryo-EM structure or the symmetry was disrupted in the subtomogram average.

### Tomogram segmentation

Tomogram segmentations were performed using Dragonfly (*16*). Hand segmentation was performed on one tomogram containing all relevant features following the protocol laid out by Heebner *et al.* (*25*). This segmentation was used to iteratively correct and train a neural net to identify features in the data including, plasma membrane, fiducials, and LPL filaments. The resulting segmentations were used to assess distance of fiducials from membranes and provide confirmation of hand-picked particle locations in both Dragonfly ORS and ChimeraX (*16*, *17*).

### bLPL purification

Bovine LPL for in vitro structure characterization was purified as described previously (*5*). Briefly, LPL was isolated from raw cow’s milk using heparin sepharose beads. LPL was eluted, buffer-exchanged, and bound to a HiPrep heparin fast flow resin. LPL was eluted using 2 M NaCl, 10 mM bis-tris (pH 6.5), and 10% glycerol.

### Negative stain TEM

nsTEM samples were prepared using 2% uranyl acetate in 50% EtOH on continuous carbon 300-mesh grids as previously described (*5*). Grids were imaged using a FEI Tecnai T12 TEM equipped with a Gatan Rio complementary metal-oxide semiconductor camera. LPL samples were prepared in different buffer conditions at 4 μM concentration. Tested buffers include: 20 mM tris-HCl (pH 7.5) and 500 mM NaCl; 20 mM Hepes (pH 7.4) and 500 mM NaCl; 20 mM tris-HCl (pH 7.2) and 500 mM NaCl; and 20 mM tris-HCl (pH 7.5) and 750 mM NaCl.

### Cryo-EM grid preparation

Cryo-EM grids were frozen using a Vitrobot Mark IV. Samples were frozen on R1.2/1.3 200 mesh Quantifoil grids, with copper support, that had a 2-nm carbon coating. Grids were glow discharged using a TERGEO on remote setting with 15-W power, 64 duty ratio, 2.5-standard cubic centimeters per minute (sccm) oxygen, and 7.5 sccm argon for 1 min. LPL stored in 2 M NaCl, 10 mM bis-tris (pH 6.5), and 10% glycerol was diluted 1:4 in 20 mM Hepes (pH 7.4) with 500 mM NaCl. The final LPL concentration in the sample was 0.8 mg/ml. Sample was applied to each grid at 4°C and 100% humidity. The sample was incubated for 10 s before blotting for 3 to 4 s and plunge freezing into a liquid ethane/propane mix.

### Cryo-EM data collection

Cryo-EM data were collected using SerialEM (*23*) with a 200-kV Talos Arctica equipped with a Gatan K3 direct electron detector. Micrographs (2952) were collected with a dose of 58 e−/Å^2^ and a pixel size of 0.876 Å/pixel.

### Cryo-EM reconstruction

Cryo-EM data were analyzed with cryoSPARC (*20*). Micrographs were curated to 2523, removing images with low-predicted CTF resolution and high motion across the image. The data were motion-corrected using patch motion correction, and the CTF was estimated using patch CTF estimation. Approximately 500 particles were picked by hand to select only the smaller diameter LPL filaments present in the sample. Following 2D classification of the manually picked particles, four classes were used as templates for template picking, yielding 251,000 particles. Multiple rounds of 2D classification resulted in 45,000 particles across multiple classes. The power spectrum of 2D class that contained particles with low tilt of the filament through the ice were extracted to a 768-pixel box and used to generate a power spectrum for determination of helical symmetry parameters. Initial symmetry was estimated as a rise = 24 Å and a twist = 64.3°. A featureless cylinder was used as the starting volume for initial reconstructions. The 3D refinement with these helical parameters and C1 symmetry yielded a promising map and revealed that the filament had D1 symmetry. Applying local CTF refinement, D1 symmetry, and nonuniform refinement (*26*) yielded a 4.2-Å map based on the gold standard Fourier shell correlation (FSC) (0.143). The hand of the reconstruction was flipped, revealing that the corrected and refined helical parameters for the filament are rise = 23.7 Å and twist = −65.95°. Sharpening was performed with a *B* factor of 220.

### Cryo-EM model building

The molecular model for the 11-nm LPL filament was created using Coot and PHENIX (*27*, *28*). First, a volume corresponding to a single LPL monomer was segmented from the cryo-EM density in ChimeraX using the volume mask tool. This map was imported into PHENIX using a monomer from Protein Data Bank (PDB) 6U7M as the initial model. Residues were added to the model in Coot to fill in the missing lid region using the Fit Loop by Rama Search tool. The monomer model coordinates were iteratively modified in Coot and refined using real-space refinement in PHENIX. The refined monomeric model was then duplicated in ChimeraX to generate a dimeric model, which was refined with the same steps as the monomer model but to match the volume data corresponding to a dihedral dimer segmented from the central region of the cryo-EM filament map. The initial filamentous model was generated by expanding the refined dimer in ChimeraX according to the calculated helical parameters. Clashes identified in the filamentous structure were corrected in one dimer that was then used to regenerate the filament model. Interface analysis was performed with PDBePISA (*29*) using an 8-nucleotide oligomer of the monomer that represented all possible contacts between the asymmetric unit (dihedral dimer) and neighboring chains.
