# Supplementary material for: Cryogenic electron tomography reveals helical organization of lipoprotein lipase in storage vesicles
Source: Sci Adv. 2025 Aug 6;11(32):eadx8711. doi: 10.1126/sciadv.adx8711 (PMC12327459; doi:10.1126/sciadv.adx8711)
Supplement: Supplementary file 1 — Figs. S1 to S9 Table S1 Legend for movie S1 [file sciadv.adx8711_sm.pdf]

Science Advances
AAAS

Supplementary Materials for

Cryogenic electron tomography reveals helical organization of lipoprotein lipase in storage vesicles

Kathryn H. Gunn *et al.*

Corresponding author: Kathryn H. Gunn, kathryn.gunn@stonybrook.edu; Saskia B. Neher, neher@email.unc.edu

Sci. Adv. **11**, eadx8711 (2025)
DOI: 10.1126/sciadv.adx8711

The PDF file includes:

Figs. S1 to S9
Table S1
Legend for movie S1

Other Supplementary Material for this manuscript includes the following:

Movie S1

Fig S1. Full Westerns for Figure 1

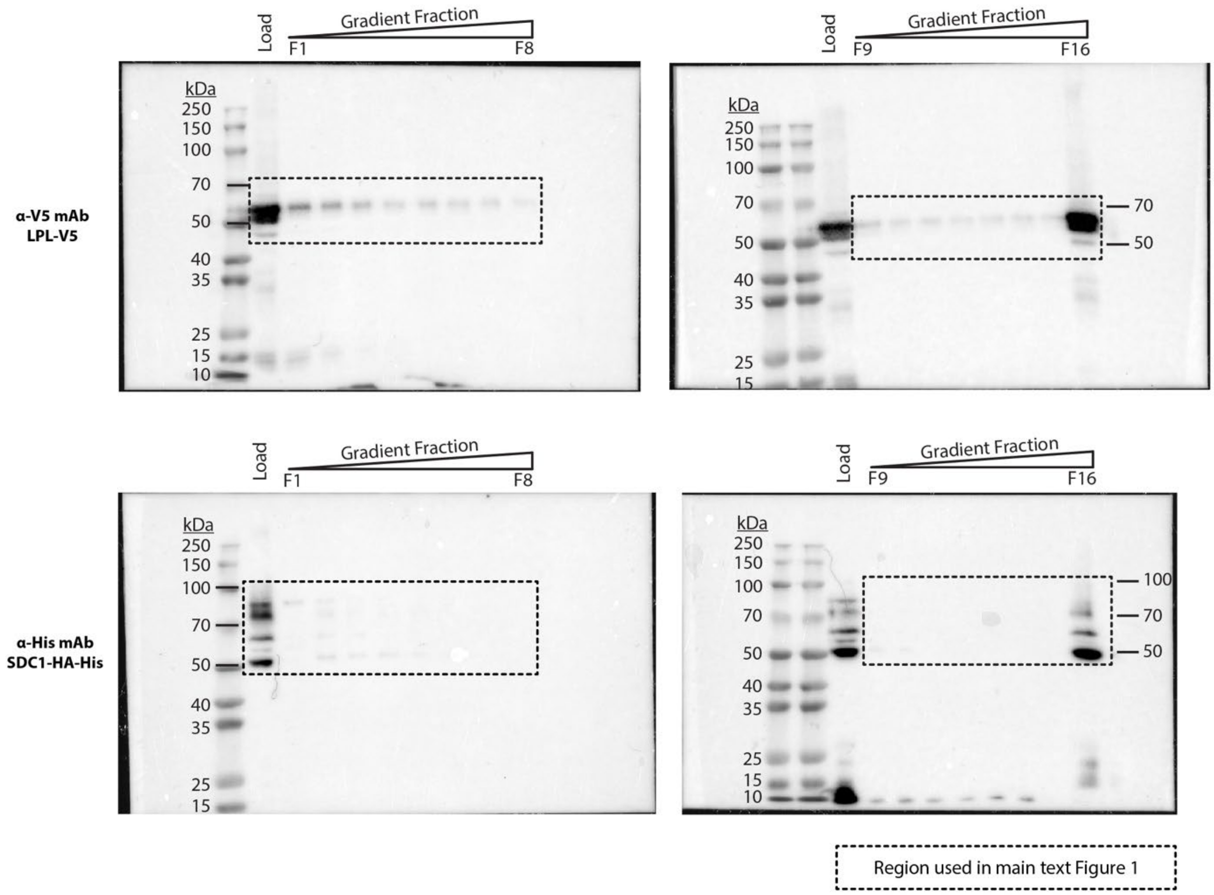

LEGEND: Western Blots of LPL and SDC-1 as separated by density gradient fractionation. The areas of the gels used in **Figure 1E-F** are boxed.

Fig S2. The vesicles analyzed for this study

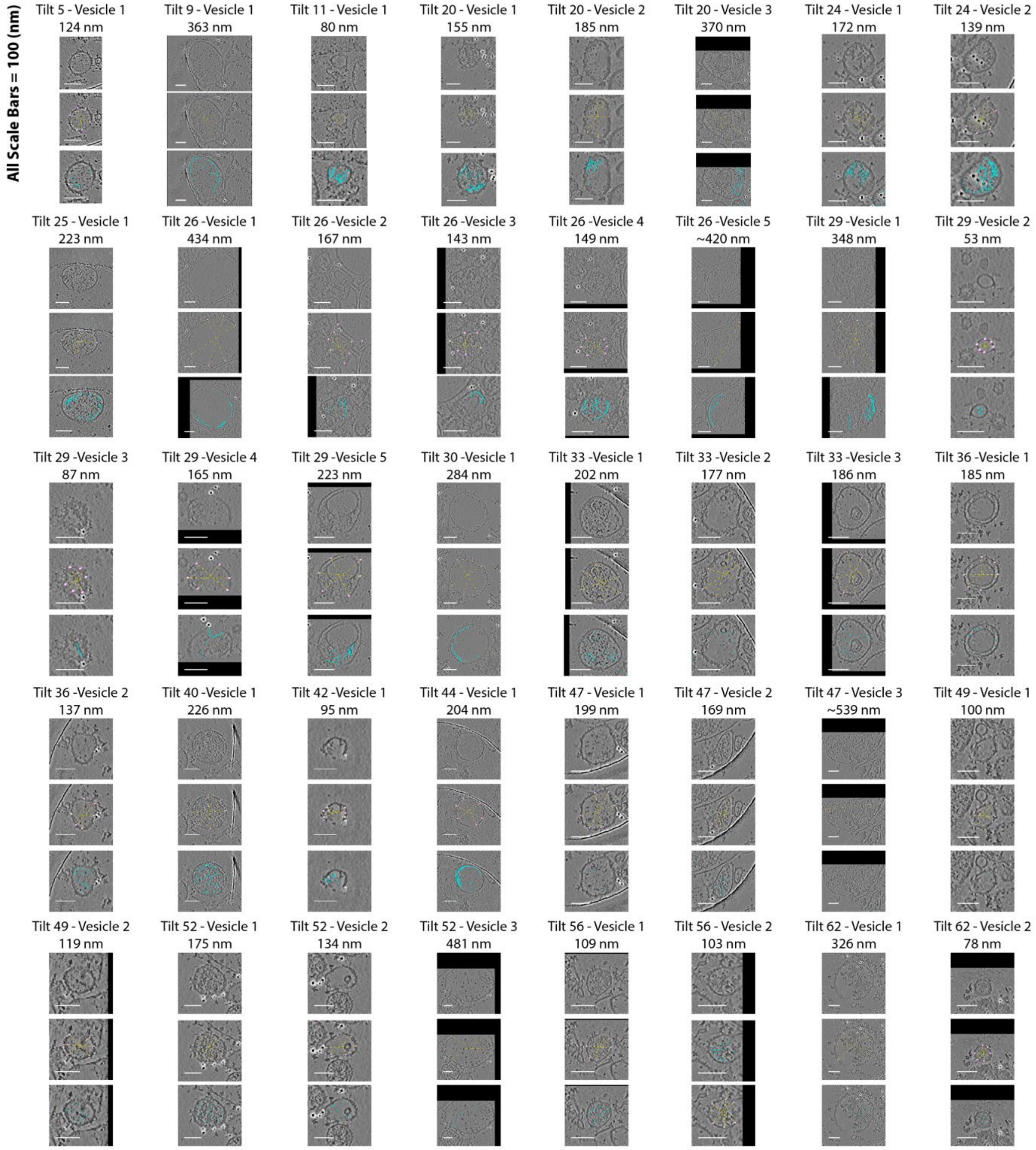

Legend: The 40 vesicles analyzed in this manuscript. Top: cryoET slice at widest part of vesicle. Middle: Three measurements to determine a rough diameter estimate of the vesicle. Bottom: The vesicle overlaid with the particle locations picked.

Fig. S3. General Subtomogram Averaging Process for Final Map

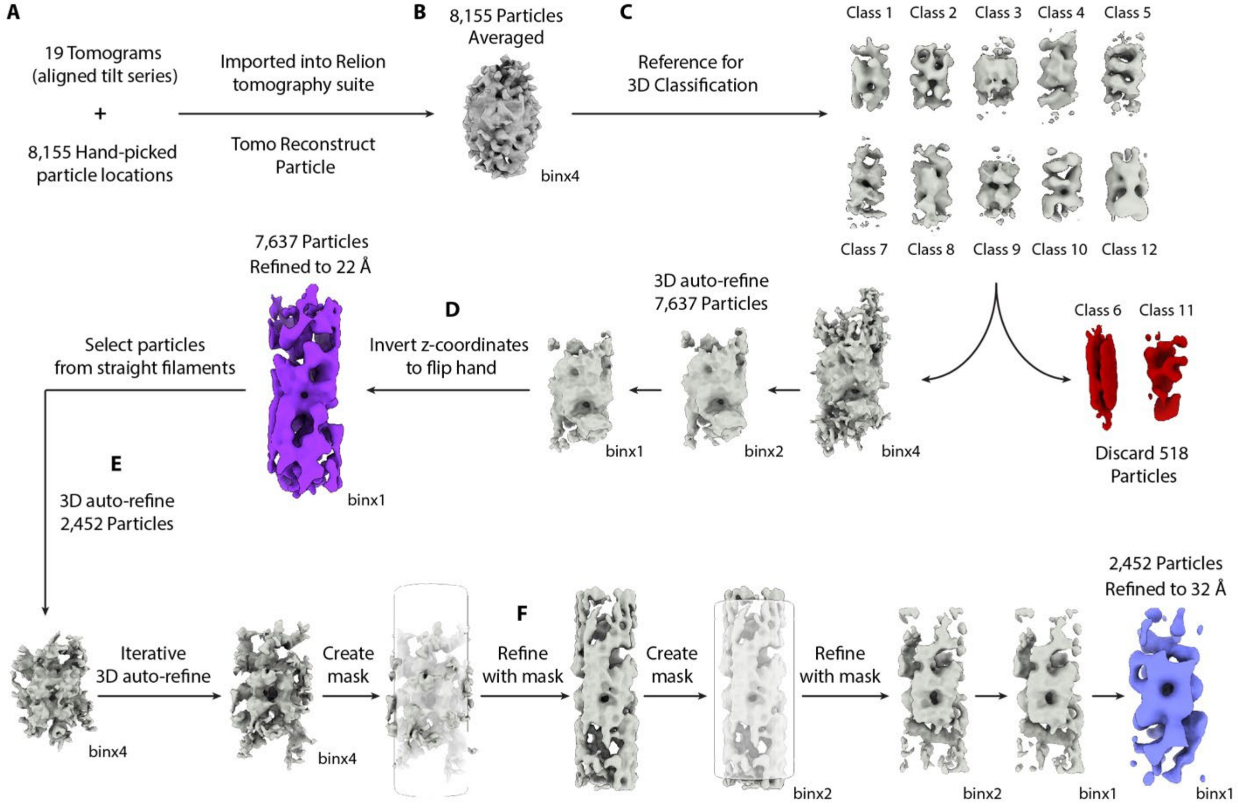

LEGEND: (A–F) See "Subtomogram Averaging" section of Methods. **(A)** Initial processing steps after tomogram collection, reconstruction, and particle selection. Particles from 19 tilt series were imported into Relion. The 8,155 particles averaged into the shape shown in (B). **(B)** Averaged particles without alignment, binned by a factor of four. **(C)** The volume from (B) was used as a reference for 3D classification. Of the 12 classes, two classes comprised of 518 particles were discarded. Class 6 appeared to be membrane bilayer and Class 11 had a lot of discontinuous density. The remaining 7,637 particles were iteratively processed with 3D auto-refine, starting with a binning factor of four, then two, and then one (unbinned). **(D)** The iterative refinement was not significantly improving the map, so we suspected the data was inverted and the volume was of the opposite hand. By flipping the sign of the z-coordinates in both the particle coordinates and in the tilt series alignments, we were able to reverse the hand of the particles and obtain a better reconstruction. **(E)** For additional improvement, particles from straight filaments (2,452 out of 7,637 particles) were separated and processed with similar steps. **(F)** A featureless cylinder that contained the inner region of the density but excluded the outer edges of the filament was used as a reference mask to focus refinement on the areas of the filament that are likely to follow a homogeneous pattern and not be interrupted by heterogeneous heparan binding. In subsequent steps, the cylindrical mask was widened to include the whole diameter of the protein components of the filament and the binning factor was decreased iteratively to obtain the final map.

Fig. S4 Imaging conditions that stabilize 11 nanometer filament formation.

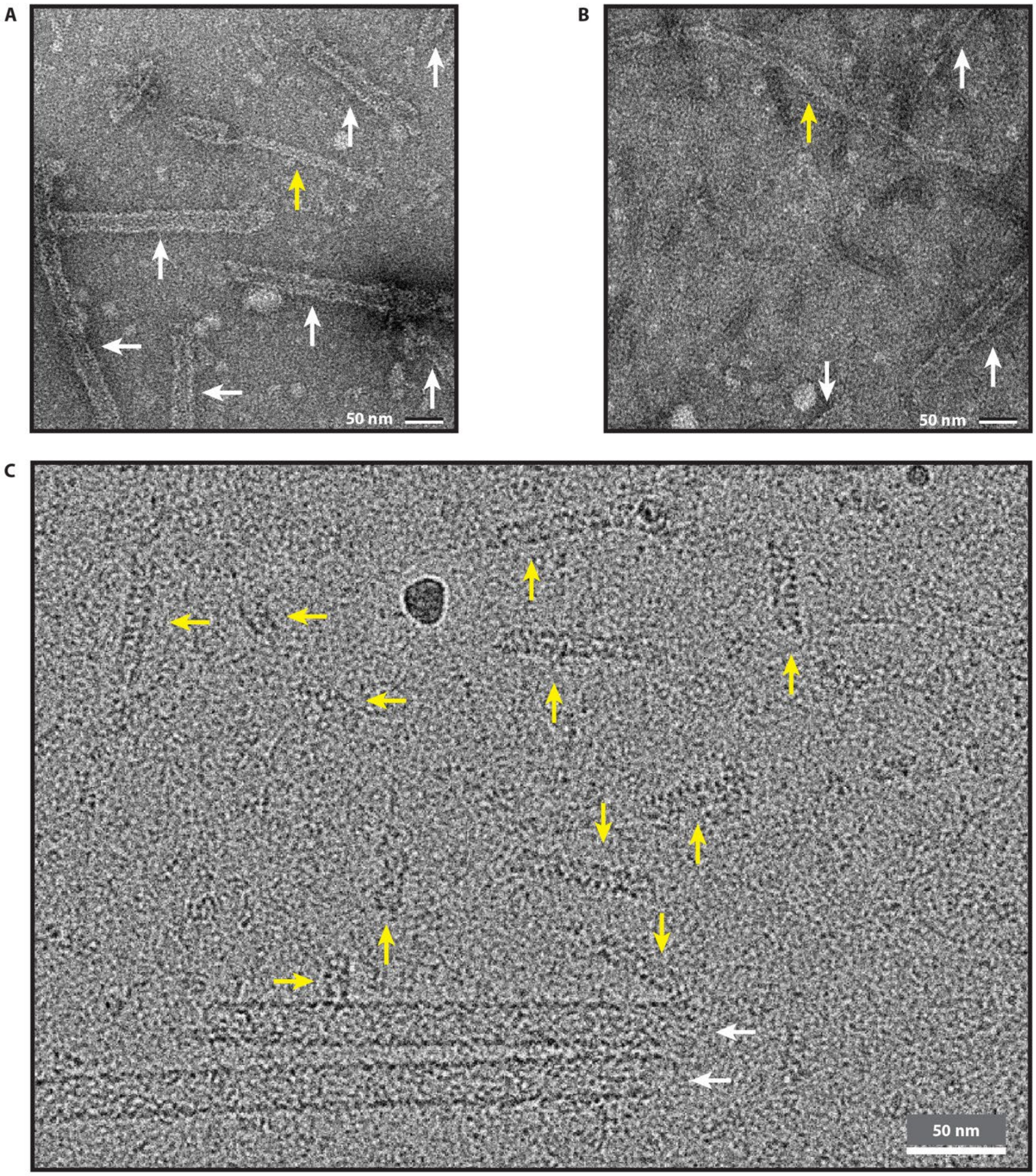

Legend: All scale bars indicate a length of 50 nanometers. Yellow arrows point to 11 nanometer LPL filaments and white arrows point to 25 nanometer LPL filaments. **(A)** nsTEM of 4 µM LPL in 20 mM Tris HCl pH 7.5, 500mM NaCl. **(B)** nsTEM of 4 µM LPL in 20mM HEPES pH 7.4, 500mM NaCl. **(C)** cryoEM of 0.8 mg/mL LPL in 20mM HEPES pH 7.4, 500mM NaCl.

Figure S5 – CryoEM data for helical symmetry determination.

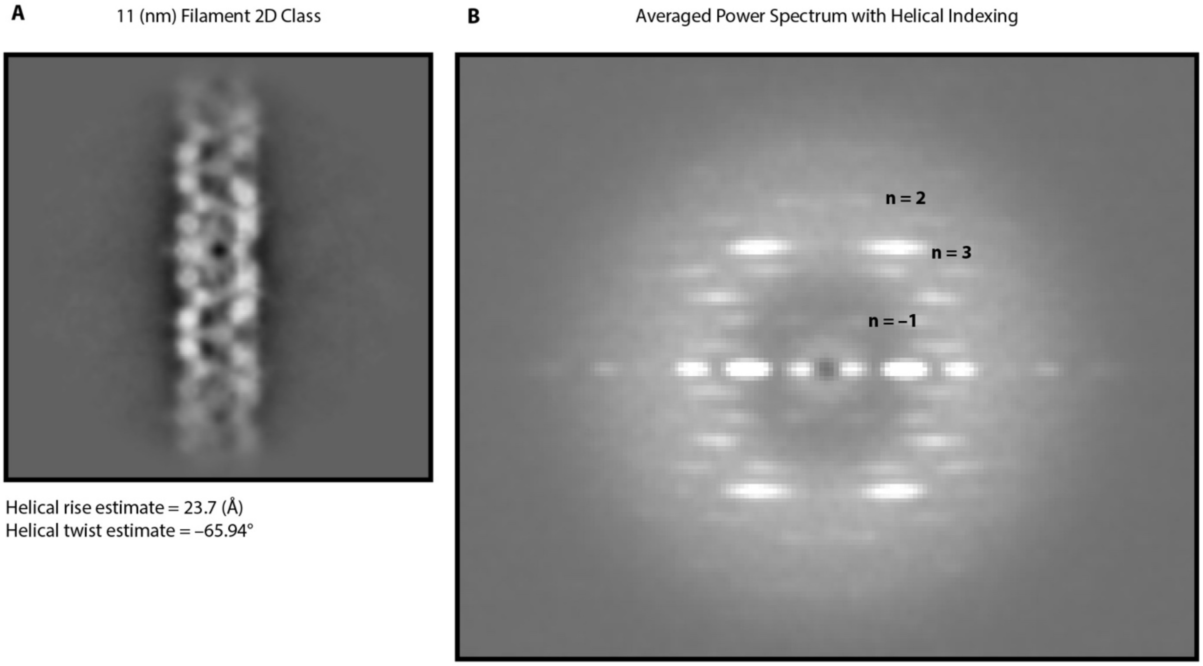

LEGEND: (A) Representative 2D class from the cryoEM dataset. **(B)** The averaged power spectrum of the 2D class overlaid with helical indexing. The helical rise was estimated at 23.7Å and the helical twist at –65.94°.

Fig. S6 – The 11 nanometer cryoEM SPA structure features low-resolution data for the LPL lid that was not previously well-resolved in other cryoEM structures

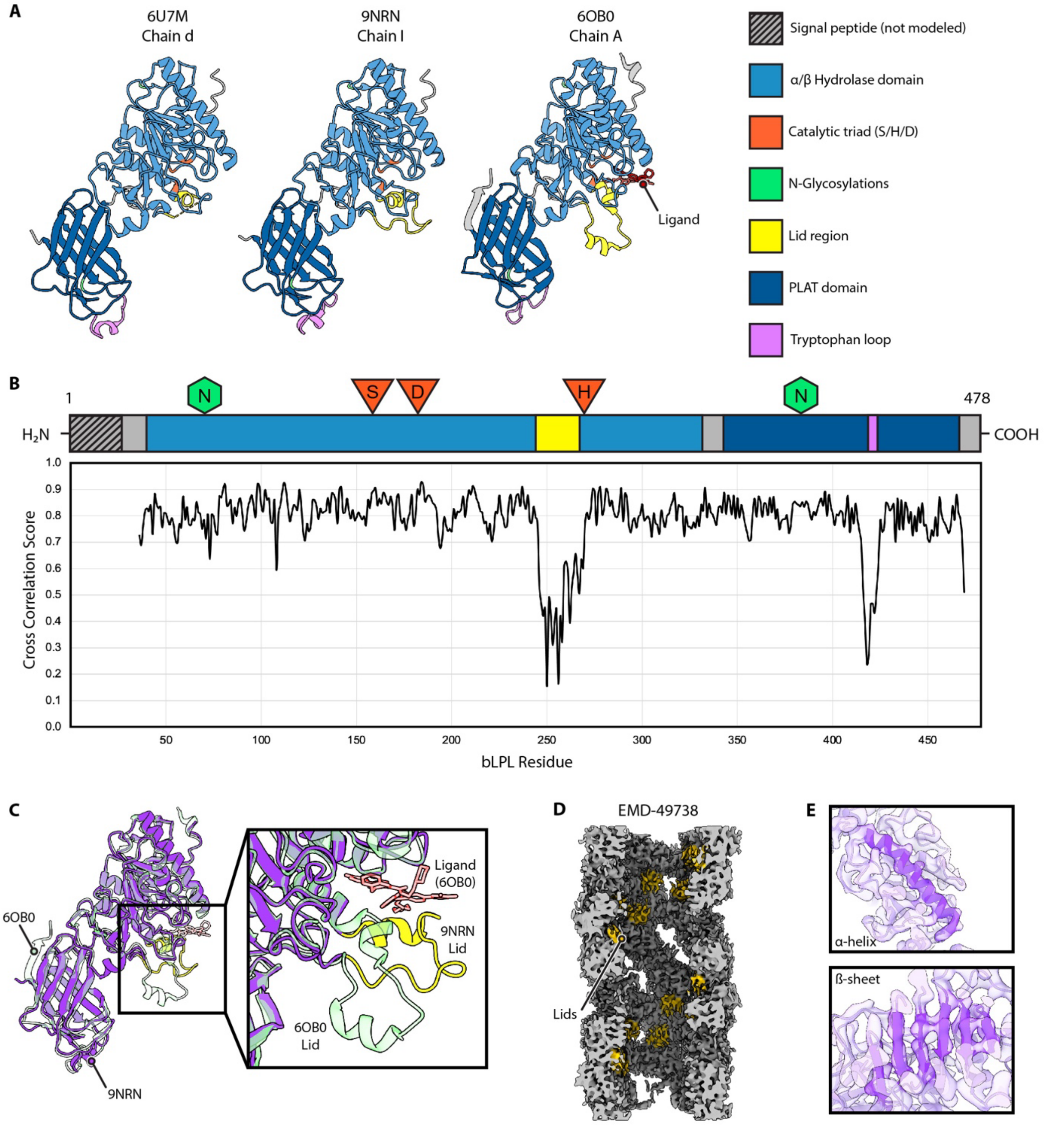

LEGEND: **(A)** Left: Ribbon representation of chain 'd' from the model of the 25 nanometer LPL filament, PDB 6U7M. This model, which lacks a lid, was the initial input model to serve as a starting point for refining the monomer model of the 11 nanometer filament, PDB 9NRN (center, chain I). Right: LPL monomer (chain A) from crystal structure of human LPL in complex with a ligand, PDB 6OB0. **(B)** Cross correlation (CC) score per residue output from PHENIX real space refinement. On top of the CC plot is a domain map of bLPL that follows the same legend as in (A). Asparagine-linked glycosylations are shown as green hexagons and the residues of the

catalytic triad are shown as orange triangles on the domain map. **(C)** Chain I from the 11 nanometer filament model (purple and yellow) overlaid with 6OB0 and its ligand (green and red transparencies). **(D)** Longitudinal cutaway of the 11 nanometer bLPL filament with the density surrounding the lid domains shown in yellow, contour level 0.0576. **(E)** Representative alpha helix (above) and beta sheet (below) of the cryoEM SPA structure of the 11 nanometer filament viewed as a transparency over the ribbon structure of the model (Chain J). Viewing contour level 0.163 in ChimeraX.

Fig. S7 –Comparison of the 25 nanometer and 11 nanometer filaments reveals nearly identical dihedral dimers with different orientations

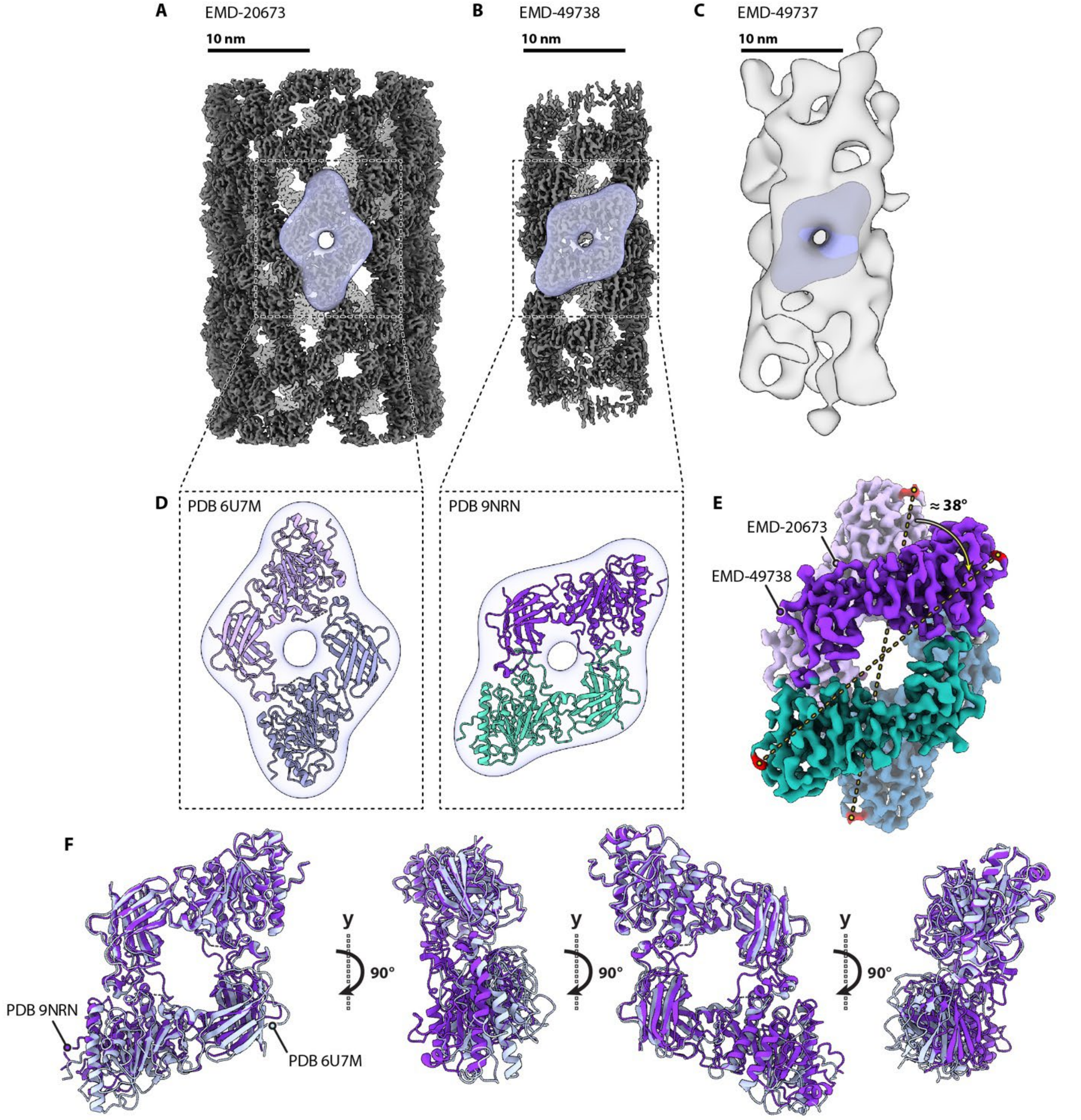

LEGEND: (A–C) Scale bar represents 10 nanometers. **(A)** EMDB 20673, the cryoEM SPA structure of the 25 nanometer bLPL filament, with a central dimer overlaid with a lowpass filtered dimer volume to more easily visualize the repeating subunits and their tilt. **(B)** The cryoEM SPA structure of the 11 nanometer bLPL filament with a lowpass filtered dimer volume overlaying a central dimer for visualization, as in (A). **(C)** CryoET STA structure obtained from filaments imaged inside vesicles. The filtered dimer volume from (B) is fitted into the central region of the filament. **(D)** Chains B and c from PDB 6U7M (wider filament) and chains I and J from the model of the thinner filament. **(E)** Volumes segmented from the 25- and 11 nanometer filaments

corresponding to dihedral bLPL dimers. There is an approximately 38° angle on the z-axis between identical residues on each pair. The volumes around residues 36 and 37 are colored in red in each dimer. The dimer in the back is from the wider filament, and the dimer in the front is from the thinner filament. **(F)** The models of the dihedral dimers from the 25 nanometer filament (chains B and c, light blue) and the 11 nanometer filament (chains I and J, purple). The four frames are the same dimers turned 90° about the y-axis three times. The model alignment was performed with the ChimeraX Matchmaker tool.

Fig. S8. Helical Interfaces of the 11 nanometer Filament

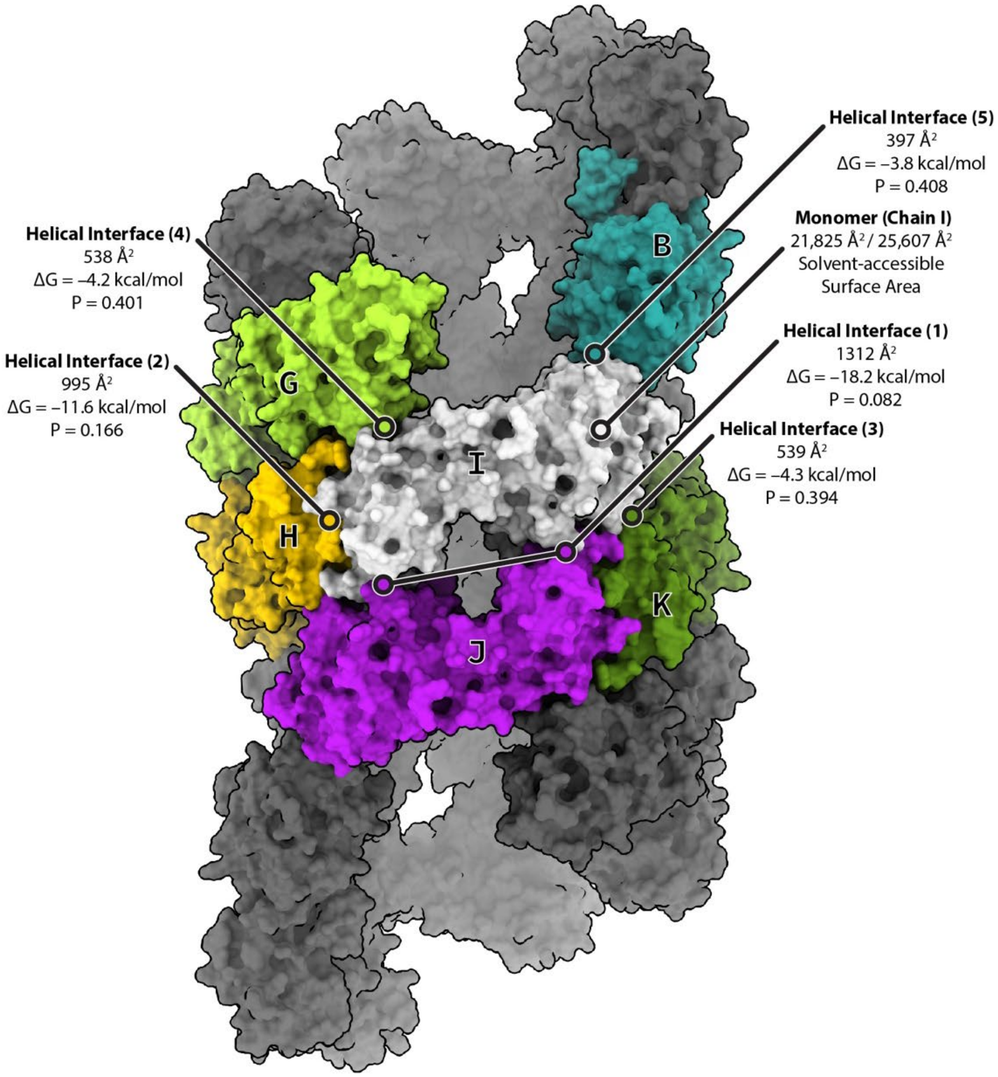

LEGEND: Five interfaces are present on each internal monomer (labeled with their respective chain IDs) in the 11 nanometer LPL filament. The interfaces are listed in order of decreasing interface area, calculated as the average of the buried surface areas of the two chains in Ångstroms squared, and increasing ΔG (solvation free energy) in kilocalories per mole, where a negative ΔG value indicates positive protein affinity through hydrophobic interactions. The ΔG P-value is the probability of getting a ΔG value lower than observed if a group of atoms with the same total surface area as the interface were picked randomly from the protein surface. The same and additional interface data are tabulated in Table S1. Interface analysis was performed using the PDBePISA tool and the model of the filament was rendered in the surface representation using ChimeraX.

Fig. S9. Local Resolution Maps of Helical Reconstruction and Subtomogram Average

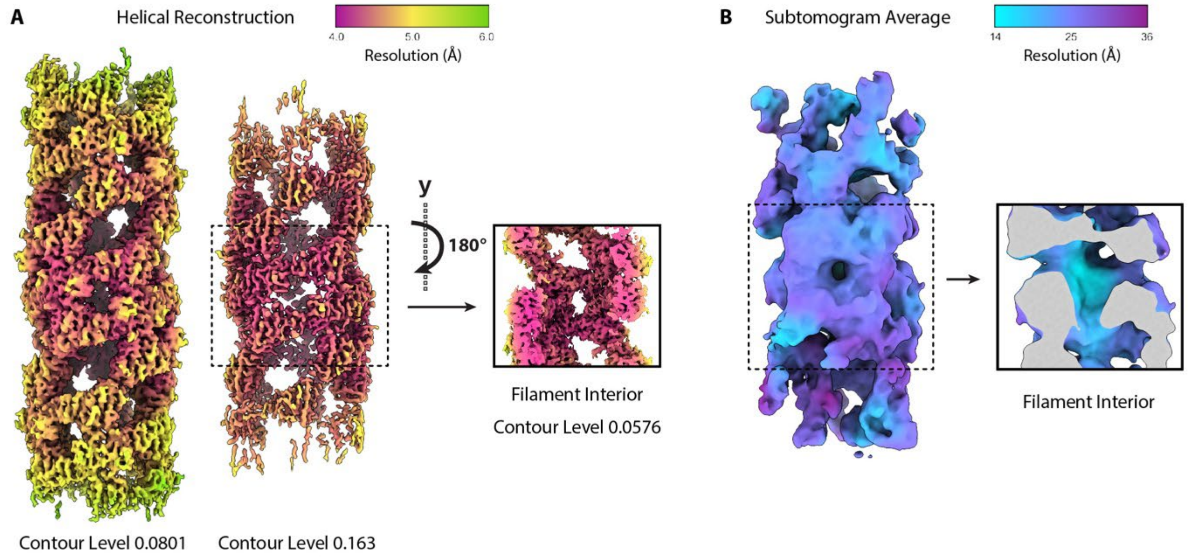

LEGEND: (A–B) Local resolution maps generated with Relion and rendered with ChimeraX. **(A)** Local resolution ranges from about 4–6 Å in the map solved by helical reconstruction with 45,000 particles. With this processing method, data farther from the center tends to be lower resolution than data closer to the center. **(B)** Local resolution ranges from about 14–36 Å in the map solved by subtomogram averaging with ~2,500 intra-vesicular particles. Some regions of higher resolution are found in the filament interior. Shown here is the map generated from post-processing (B factor of 1000) with no lowpass filter.

Table S1 – PDBPISA Interface Analysis of the 11 nanometer Filament

Protein interfaces, surfaces and assemblies (PISA) tool, available from the European Bioinformatics Institute (*1, 2*) was used for the interface analysis presented in this table. **(A)** Summary of unique interfaces on each internal monomer in the 11 nanometer bLPL filament. Interface area is calculated as the average of the buried surface areas of the two chains in Ångstroms squared. See Supplemental Figure 6 for a pictorial representation. Interface ΔG is the solvation free energy gain upon interface formation and is measured in kilocalories per mole, where a negative ΔG value indicates positive protein affinity through hydrophobic interactions. The ΔG P-value is the probability of getting a ΔG value lower than observed if a group of atoms with the same total surface area as the interface were picked randomly from the protein surface. The numbers of hydrogen bonds (NHB), salt bridges (NSB), and disulfide bridges (NDS) per interface (from both chains combined) are indicated, as well as the number of residues per chain in each interface. **(B)** The solvent-accessible surface areas (ASA) of residues in a bLPL monomer in isolation (uncomplexed) and the accessible surface areas buried by filament formation, both in Ångstroms squared. Chain I is used as a representative internal monomer of the filament that makes all possible contacts with other monomers. Residues participating in hydrogen bonds are in bold and marked with an asterisk (*). Total accessible and interface-buried surface areas on Chain I are listed at the bottom of the table, and these values differ slightly from the averaged Interface Area values listed in (A). Table includes only residues participating in an interface, but total ASA value is the sum of ASA for all residues in chain I (Asp-36 to His-469), including those not listed here.

A. Summary of Filament Interface Analysis

	Chains	Interface Area (Å^2)	ΔG (kcal/mol)	ΔG P-value	NHB / NSB / NDS	# Residues (Chain I / Other Chain)
1	I/J	1312	-18.2	0.082	0 / 0 / 0	42 / 42
2	I/H	995	-11.6	0.166	6 / 0 / 0	28 / 27
3	I/K	539	-4.3	0.394	1 / 0 / 0	17 / 18
4	I/G	538	-4.2	0.401	1 / 0 / 0	18 / 17
5	I/B	397	-3.8	0.408	0 / 0 / 0	15 / 15

B. Residues of Chain I and Buried Surface Areas in Filament Interfaces

	bLPL Residue (Chain I)	Isolated ASA (Å^2)	Surface Area (Å^2) Buried by Interface				
			1 (I/J)	2 (I/H)	3 (I/K)	4 (I/G)	5 (I/B)
1	63 VAL	80.4	--	--	--	--	2.5
2	64 THR	61.1	--	--	--	--	49.6
3	65 GLU	113.9	--	--	--	--	72.4
4	67 VAL	11.0	--	--	--	--	1.0
5	68 ALA	89.3	--	--	--	--	60.7
6	69 ASN	103.5	--	--	--	--	5.8
7	71 HIS	173.8	--	--	--	--	39.4
8	72 PHE	14.0	--	--	--	--	1.3
9	73 ASN	55.0	--	--	--	--	0.3
10	74 HIS	65.7	--	--	--	--	47.3
11	85 TRP	94.3	59.8	--	15.9	--	--
12	87 VAL	92.8	21.6	--	42.9	--	--

13	88	THR	70.0	--	--	22.2	--	--
14	89	GLY	48.4	--	--	0.6	--	--
15	121	GLN	79.5	--	--	40.4	--	--
16	122	GLN	106.5	--	--	13.9	--	--
17	123	HIS	125.8	31.6	--	89.6	--	--
18	124	TYR	50.4	28.4	--	3.4	--	--
19	125	PRO	61.9	13.6	--	--	--	--
20	126	VAL	47.2	--	--	15.0	--	--
21	148	GLU	100.6	--	--	--	--	35.1
22	149	PHE	54.8	--	--	--	--	52.5
23	150	ASN	115.4	--	--	--	--	29.5
24	151	TYR	7.2	--	--	--	--	0.5
25	152	PRO	69.7	--	--	--	--	1.5
26	162	SER	8.6	5.4	--	--	--	--
27	163	LEU	5.7	1.8	--	--	--	--
28	190	PRO	31.6	21.4	--	--	--	--
29	191	ASN	103.1	0.4	--	--	--	--
30	215	PHE	49.8	9.0	--	--	--	--
31	217	ARG	140.5	32.3	--	--	--	--
32	222	ARG	197.5	89.3	--	--	--	--
33	224	ILE	65.1	47.5	--	--	--	--
34	248	ILE	163.6	15.7	--	--	--	--
35	250	GLU	41.5	32.3	--	--	--	--
36	251	ALA	82.1	7.5	--	--	--	--
37	252	LEU	95.8	27.6	--	--	--	--
38	253	ARG	160.5	67.8	--	31.6	--	--
39	254	VAL	119.8	--	--	69.5	--	--
40	258	ARG	104.6	26.3	--	57.6	--	--
41	**259**	**GLY***	47.1	11.7	--	34.0	--	--
42	260	LEU	112.6	--	--	107.1	--	--
43	261	GLY	37.0	--	--	7.2	--	--
44	262	ASP	110.1	--	--	3.4	--	--
45	264	ASP	30.2	14.1	--	--	--	--
46	267	VAL	61.8	32.5	--	--	--	--
47	268	LYS	86.0	41.5	--	--	--	--
48	271	HIS	20.2	7.6	--	--	--	--
49	355	THR	133.3	--	3.0	--	--	--
50	370	TYR	54.8	--	--	--	46.4	--
51	371	GLY	20.6	--	--	--	20.6	--
52	372	THR	88.2	--	--	--	71.0	--
53	373	VAL	101.6	--	--	--	19.3	--
54	374	ALA	31.1	--	--	--	5.0	--
55	375	GLU	73.4	--	--	--	23.4	--
56	377	GLU	102.5	--	--	--	16.2	--

57	378	ASN	119.4	32.3	--	--	--	--
58	404	GLU	92.8	--	--	--	47.2	--
59	406	LEU	98.5	--	31.1	--	39.6	--
60	407	MET	62.7	--	--	--	48.7	--
61	409	LYS	40.9	--	--	--	2.2	--
62	411	LYS	81.1	46.2	--	--	--	--
63	413	ILE	64.0	1.0	--	--	--	--
64	415	ASP	103.2	39.3	--	--	--	--
65	416	SER	41.5	0.6	--	--	--	--
66	417	TYR	171.6	102.6	--	--	--	--
67	418	PHE	136.1	110.3	11.1	--	--	--
68	419	SER	69.2	20.0	23.1	--	--	--
69	420	TRP	182.1	155.6	--	--	--	--
70	421	SER	20.3	--	1.8	--	--	--
71	**422**	**ASN***	98.3	--	58.8	--	--	--
72	423	TRP	226.8	42.8	85.1	--	--	--
73	424	TRP	134.6	68.7	--	--	--	--
74	425	SER	102.6	17.4	--	--	--	--
75	426	SER	12.9	0.2	0.2	--	--	--
76	427	PRO	47.5	9.5	--	--	--	--
77	443	LYS	78.0	--	--	--	18.9	--
78	444	LYS	124.6	--	--	--	0.6	--
79	446	ILE	36.9	--	--	--	0.7	--
80	448	CYS	20.9	--	11.5	--	--	--
81	449	SER	20.7	--	5.7	--	--	--
82	**450**	**ARG***	130.3	--	109.3	--	--	--
83	451	GLU	144.2	--	49.5	--	--	--
84	452	LYS	171.9	--	57.3	--	--	--
85	453	MET	152.0	--	139.2	--	--	--
86	454	SER	16.3	--	10.2	--	--	--
87	455	TYR	134.3	--	87.8	--	--	--
88	**457**	**GLN***	37.9	--	29.9	--	--	--
89	458	LYS	47.0	--	2.8	--	--	--
90	459	GLY	43.6	2.3	6.9	--	--	--
91	460	LYS	112.9	4.9	97.3	--	--	--
92	461	SER	73.9	8.4	35.3	--	--	--
93	462	PRO	32.3	0.3	5.4	--	--	--
94	463	VAL	5.7	--	4.0	--	--	--
95	464	ILE	70.2	--	46.7	--	5.7	--
96	466	VAL	50.9	--	38.5	--	--	--
97	467	LYS	150.9	--	16.6	--	89.9	--
98	468	CYS	46.1	--	25.4	--	14.1	--
99	469	HIS	172.3	--	1.5	--	54.7	--
**TOTAL:**			**21825**	**1309**	**995**	**555**	**524**	**399**

Movie S1.

Slices through the planes of tomogram (tilt series) number 25 showing locations of filament selections in a vesicle. Scale bar depicts a length of 20 nanometers. Shown first in the XY plane without labeling, moving through the vesicle top to bottom and then back again; next shown with filament locations labeled in purple. Next, the same vesicle shown in the XZ plane (rotated 90 degrees about the x-axis); first unlabeled and then with the same filaments labeled in purple. The high contrast circular objects are gold labels on the vesicle surface. Tomogram, filaments, and movie rendered in ArtiaX add-on for ChimeraX.
